# Eugenic and Euthenic Notes

**Published:** 1916-01

**Authors:** 


### Eugenic and Euthenic Notes.

*Heredity and Environment.*—Only by constant selection of the best can any race ever be improved. No education, no environment of any nature, can ever make any appreciable progress, even though these same favorable surroundings may produce through ages a definite but indefinitely slow increment which by constant repetition becomes slowly available in heredity, but by no means fixed, so that production, true to the better type, can be depended on.—*Luther Burbank.*

*Playgrounds vs. Schoolhouses.*—Once upon a time the citizens of a certain city in Greece were greatly interested in the nurture and training of children. When the question arose as to whether they should build a great public school or an open-air playground, it was decided to open a playground. Now, in the lapse of years, it came to pass that the citizens of that city advanced so far beyond the rest of the human race that in all the centuries since, even to this day, the nations that have gone on building schools and neglecting to open playgrounds have not been able to catch up with them.—*Geo. E. Johnson.*

*Neurasthenia.*—A fact of importance which we may mention is that neurasthenia and other neurotic conditions are apparently becoming much less common, in spite of the anxiety and strain resulting from the war. This is not difficult to understand, as the experience of most people who have been accustomed to the treatment of nervous conditions indicates that it is not so much the great tragedies of life which are apt to upset the equilibrium of the nervous system but small daily worries persisting for long periods of time, and, above all, lack of occupation and interest in life.—*Dr. W. H. B. Aikens.*

*Applied Physiology.*—The gains in human vitality which we can secure from applied physiology will, I believe, prove as surprising as those we are already securing from applied bacteriology. To this end we need to formulate scientifically what constitutes a wholesome life and to educate the people on what changes from existing customs are necessary to live that life. Furthermore, in the industrial world, we must in some cases compel wholesome mental and physical conditions for our working people. The time has come when science must subdue customs and reason triumph over tradition.—*Irving Fisher.*

*Law of the Stoics.*—"There is a way of looking at our poverty, our plainness of feature, our lack of mental brilliancy, our humble estate, our unpopularity, our physical ailments, which, instead of making us miserable, will make us modest, contented, cheerful, serene. The mistakes that we make, the foolish words we say, the unfortunate investments into which we get drawn, the failures we experience, all may be transformed by the Stoic formula into spurs to greater effort and stimulus to wiser deeds in days to come. Simply to shift the emphasis from the dead external fact beyond our control to live option which always presents itself within; and to know that the circumstance that can make us miserable simply does not exist, unless it exists by our own consent within our own minds; this is a lesson well worth spending an hour with the Stoics to learn once for all."—*President Hyde.*

